# Functional Outcomes Following Arthroscopic Anterior Cruciate Ligament Reconstruction (ACLR) With and Without Internal Bracing: A Prospective Study

**DOI:** 10.7759/cureus.98455

**Published:** 2025-12-04

**Authors:** Rishav Choudhary, Atul Agrawal, Faiz Akbar Siddiqui

**Affiliations:** 1 Department of Orthopaedics, Himalayan Institute of Medical Sciences, Dehradun, IND

**Keywords:** acl reconstruction, anterior cruciate ligament, functional outcome, internal bracing, lysholm score

## Abstract

Background and objective

Internal bracing (IB) is a relatively new technique in anterior cruciate ligament reconstruction (ACLR), proposed to improve graft protection during early healing. This study aimed to compare functional outcomes of ACLR with and without IB.

Methods

A prospective observational study was conducted over 12 months at a tertiary care hospital. Fifty patients with ACL injuries were divided into two groups: Group A (n = 25) underwent arthroscopic ACLR with IB, and Group B (n = 25) without IB. The patients were assigned to the two groups based on their informed choice, ensuring balanced group distribution until the sample size was achieved. Baseline demographics, injury profiles, graft parameters, and Lysholm Knee Scores were recorded preoperatively and at 6 and 12 weeks postoperatively. Statistical analysis was performed using IBM SPSS Statistics for Windows, Version 26 (Released 2018; IBM Corp., Armonk, NY, USA), with a p-value less than 0.05 considered significant.

Results

The mean age was higher in Group A (32.92 ± 10.91) than in Group B (26.56 ± 8.66; p = 0.027). ACL tears were present in all Group B and 84% of Group A patients. Both groups had similar sociodemographic, clinical, and arthroscopic profiles. Group B had longer semitendinosus grafts (p = 0.007), but final graft dimensions were comparable. Lysholm scores were higher in Group A at all follow-up points (pre-op: 29.76 vs. 29.12; 6 weeks: 56.08 vs. 55.88; 12 weeks: 88.24 vs. 86.12), though not statistically significant. Minor complications, like pain, stiffness, and swelling, occurred equally across groups.

Conclusion

ACL reconstruction with IB showed comparable early functional outcomes without increasing short-term complication rates. However, larger, long-term studies are required.

## Introduction

The anterior cruciate ligament (ACL) is the most commonly injured knee ligament, responsible for around 50% of all knee ligament injuries with an annual incidence of 68.6 per 100,000 person-years [1]. In the United States alone, approximately 400,000 ACL reconstructions (ACLRs) are performed annually, representing a significant socioeconomic burden, particularly among working-age individuals [2].

ACL injuries are more prevalent in female athletes, likely due to anatomical and hormonal differences. Sports like soccer show the highest incidence [3]. Responses to ACL injuries vary - some cases heal conservatively, while severe or unstable injuries often require surgery, often with involvement of the meniscus. High-impact sports, such as basketball and football, are particularly associated with ACL injury risk, often leading to instability, pain, and restricted mobility [4].

Surgical approaches to ACL injuries have evolved over time. While open ACL repairs were once standard, modern ACLR typically uses autologous hamstring or patellar tendon grafts [4]. Traditional ACL repair has fallen out of favor due to high failure and revision rates - up to 24% - prompting a shift toward reconstruction techniques [2]. Despite this, preservation strategies for torn ACLs are gaining attention, showing encouraging functional outcomes in select cases [5].

Heusdens et al. observed two retears among 42 patients after arthroscopic ACL repair with internal bracing (IB), both requiring ACLR [6]. DiFelice et al. noted one failure among 11 repairs but provided limited follow-up data [7]. ACLR remains the gold standard for revision after failed repairs, though supporting evidence is still limited [5].

Autografts (from the patient) and allografts (from a donor) are commonly used in ACLR. Each has its benefits and limitations; autografts carry donor-site morbidity risks, while allografts may fail more frequently in younger patients [8]. Graft choice depends on patient age, activity level, and preferences [9]. However, even with optimal graft choice and timing, the risk of reinjury persists. Paterno et al. reported a higher retear risk post-ACLR than in uninjured populations [10], and Gifstad et al. found poorer functional outcomes in ACLR versus repair [11].

IB has recently emerged as a technique to reinforce reconstructed ligaments, aiming to enhance early-phase stability and reduce reinjury risks [12]. Current clinical evidence indicates that IB augmentation may reduce rerupture rates in some cohorts, although the overall literature remains limited and heterogeneous. This study aims to compare the functional outcomes of ACLR with and without IB, to guide surgical decision-making in ACL injury management.

## Materials and methods

Study setting, design, and duration

The study was conducted over 12 months in the Department of Orthopaedics at the Himalayan Institute of Medical Sciences, Dehradun, India (approval no. SHRU/HIMS/ETHICS/2025/82). Ethical clearance and informed consent were obtained prior to patient enrollment. This prospective observational study included two patient groups undergoing arthroscopic ACLR. Patients were allocated based on informed choice (non-random, patient-preference allocation). The use of patient-choice allocation may introduce selection bias because patients opting for IB may differ in expectations, activity level, or socioeconomic factors. Group A comprised patients who received ACL reconstruction with IB, while Group B included those who underwent the procedure without IB.

Inclusion and exclusion criteria

Participants included in the study were adults aged 18 years or older who presented with an ACL injury within 12 weeks and were suitable candidates for surgery. Only those capable of understanding the study and providing informed written consent were enrolled. Patients were excluded if they had a history of prior surgery or injury to the affected knee, concurrent injuries to the posterior cruciate ligament (PCL) or medial collateral ligament (MCL), associated fractures, active infections, or known allergies to implant materials. Individuals with cognitive impairments or significant medical comorbidities that could interfere with surgery or affect study outcomes were also excluded.

Sample size calculation

Sample size was calculated using the formula for comparing two means based on the Lysholm Knee Score, with α = 0.05, power = 80%, σ = 5.52, and δ = 10. This yielded n ≈ 5 per group. Accounting for dropouts and variability, 25 patients per group were included. This study was not powered to detect small differences and therefore can only exclude large early functional effects.

Study tools and data collection

Data collection for the study was facilitated using a structured case recording form, the Lysholm Knee Scoring Scale [13], and radiographic imaging, including bilateral knee anteroposterior and lateral views, as well as specific views of the affected knee. Collected data included baseline demographic details, type of injury, and preoperative functional status, assessed using the Lysholm score and radiographs. Postoperative follow-up evaluations were conducted at pre-op, 6 weeks, and 12 weeks to assess functional outcomes, including the Lysholm score, time to return to work, rupture-to-surgery interval, and any complications.

Surgical procedure

All patients first underwent diagnostic arthroscopy through an anterolateral viewing portal to confirm the ACL tear and assess intra-articular structures. Hamstring tendons - gracilis and semitendinosus - were harvested using established anatomical landmarks, and the grafts were prepared by removing excess muscle tissue and measuring their diameter. Femoral tunnels were created using an accessory medial portal, and the prepared grafts were passed and fixed with a suspensory loop on the femoral side and a suture disc or interference screw on the tibial side. In Group A, a #2 FiberTape® (Arthrex, Naples, FL, USA) was passed parallel to the graft through the femoral and tibial tunnels, and fixed using a cortical button on the femoral side and a knotless anchor on the anterior tibial cortex.

Postoperative care and outcome assessment

Postoperatively, all patients were managed with knee bracing, ice pack application, and limb elevation for the initial 48 hours to reduce swelling and provide support. Intravenous antibiotics were administered for 24 hours, followed by a three-day course of analgesics and nonsteroidal anti-inflammatory drugs. Patients underwent a structured rehabilitation program that included both open-chain and closed-chain exercises to promote recovery and restore knee function. Functional outcomes were assessed using the Lysholm Knee Scoring Scale, a validated tool ranging from 0 to 100, where higher scores indicate better knee function. This score evaluates eight parameters, including limp, support, locking, instability, pain, swelling, stair climbing, and squatting. Assessments were conducted preoperatively and at 6 and 12 weeks postoperatively. Scores were recorded during follow-up visits, and the mean values were compared between the two groups to determine the effect of IB on recovery.

Statistical analysis

Statistical analysis was performed using IBM SPSS Statistics for Windows, Version 26 (Released 2018; IBM Corp., Armonk, NY, USA), after data entry in Microsoft Excel (Microsoft® Corp., Redmond, WA, USA). Categorical variables were summarized as frequencies and percentages and analyzed using the Chi-square test. Continuous variables were expressed as mean ± standard deviation and compared using the unpaired t-test. A p-value of less than 0.05 was considered statistically significant.

## Results

The mean age was significantly higher in Group A (32.92 ± 10.91 years) compared to Group B (26.56 ± 8.66 years; p = 0.027), which may influence rehabilitation expectations and outcomes. Males predominated in both groups (60% in Group A and 84% in Group B), though the gender difference was not statistically significant (p = 0.057). Most patients were businessmen (32% in Group A and 36% in Group B), followed by working professionals, students, housewives, and sportspersons. Occupational distribution was similar between groups (p = 0.900) (Table 1).

**Table 1:** Age, gender, and occupation details of enrolled patients *indicates p < 0.05 as statistically significant

Variable	Domain	Group A		Group B		p-value
		N	%	N	%	
Age	Mean age	32.92	±10.91	26.56	±8.66	0.027*
Gender	Male	15	60	21	84	0.057
	Female	10	40	4	16	
Occupation	Business	8	32	9	36	0.900
	Working professional	7	28	5	20	
	Student	5	20	7	28	
	Housewife	3	12	3	12	
	Sports person	2	8	1	4	

The most common mode of injury in both groups was slip and fall (60% in Group A and 52% in Group B), followed by fall from a vehicle, vehicle collision, trauma, and knee twisting. Differences in injury mechanisms were not statistically significant (p = 0.188). ACL tear was present in 84% of Group A and in all patients in Group B. In Group A, 4% had tibial bony avulsion, and 12% had associated meniscal tears. The pattern of associated injuries showed no significant difference between the groups (p = 0.113) (Table 2).

**Table 2:** Mode of injury and associated injuries observed among patients ACL, anterior cruciate ligament

Variable	Domain	Group A		Group B		p-value
		N	%	N	%	
Mode of injury	Slip and fall	15	60	13	52	0.188
	Fall form vehicle	8	32	4	16	
	Vehicle collision	2	8	4	16	
	Trauma	0	0	2	8	
	Twisting of the knee	0	0	2	8	
Associated injuries	ACL tear	21	84	25	100	0.113
	ACL bony avulsion (tibial end)	1	4	0	0	
	ACL tear with meniscus tear	3	12	0	0	

The anterior drawer test was positive in all cases in both groups, while the posterior drawer test was negative in all cases in both groups. Lachman test was positive in 32% and 44% of patients in Groups A and B, respectively. The McMurray test (medial) was positive in 28% and 32% of patients in Groups A and B, respectively. The McMurray test (lateral) was positive in 24% and 32% of patients in Groups A and B, respectively. These differences were not statistically significant, with p-values of 0.280, 0.500, and 0.377, respectively (Table 3).

**Table 3:** Comparison of clinical test outcomes between Group A and Group B

Clinical tests	Outcome	Group A		Group B		p-value
		N	%	N	%	
Anterior drawer test	Positive	25	100	25	100	NA
	Negative	0	0	0	0	
Posterior drawer sign	Negative	25	100	25	100	NA
	Positive	0	0	0	0	
Lachman test	Negative	17	68	14	56	0.280
	Positive	8	32	11	44	
McMurray test (medial)	Negative	18	72	17	68	0.500
	Positive	7	28	8	32	
McMurray test (lateral)	Negative	19	76	17	68	0.377
	Positive	6	24	8	32	

ACL injury patterns showed no significant difference between groups (p = 0.395). In Groups A and B, respectively, avulsion tears were observed in 4% and 0%, complete tears in 56% and 52%, full-thickness tears in 24% each, grade 2 tears in 8% and 0%, partial tears in 4% and 16%, and unspecified tears in 4% and 8%. The PCL was intact in all patients. Medial meniscus findings were similar between groups (p = 0.482), with intact menisci in 72% (Group A) and 68% (Group B), posterior horn tears in 16% and 20%, general tears in 4% and 8%, bucket handle tears in 8% and 0%, and a full-thickness radial tear in 0% and 4%. For the lateral meniscus (p = 0.060), 76% (Group A) and 72% (Group B) were intact. Posterior horn tears were noted in 12% and 4%, other tears in 0% and 16%, torn segments in 0% and 8%, and fraying in 8% and 0%, respectively. Arthritic changes were seen in 36% of Group A and 56% of Group B patients (p = 0.156). Loose bodies were found in 4% of Group B, and synovial effusion in 4% of Group A, with no significant group differences (p = 0.312 for both) (Table 4).

**Table 4:** Comparison of arthroscopic findings between Group A and Group B ACL, anterior cruciate ligament; PCL, posterior cruciate ligament

Arthroscopic finding		Group A		Group B		p-value
		N	%	N	%	
ACL	Avulsion tear	1	4	0	0	0.395
	Complete tear	14	56	13	52	
	Full-thickness tear	6	24	6	24	
	Grade 2 ACL tear	2	8	0	0	
	Partial ACL tear	1	4	4	16	
	Torn	1	4	2	8	
PCL	Intact	25	100	25	100	NA
Medial meniscus	Intact	18	72	17	68	0.482
	Posterior horn tear	4	16	5	20	
	Torn	1	4	2	8	
	Bucket handle tear	2	8	0	0	
	Full-thickness radial tear of the posterior horn	0	0	1	4	
Lateral meniscus	Intact	19	76	18	72	0.060
	Posterior horn tear	3	12	1	4	
	Tear	0	0	4	16	
	Torn	0	0	2	8	
	Fraying	2	8	0	0	
Cartilage	Arthritic changes	9	36	14	56	0.156
	No arthritic changes	16	64	11	44	
Loose bodies	Absent	25	100	24	96	0.312
	Present	0	0	1	4	
Synovial structure	Effusion present	1	4	0	0	0.312
	Normal	24	96	25	100	

The mean length of the semitendinosus graft harvested was significantly greater in Group B (27.28 ± 0.98 cm) compared to Group A (26.44 ± 1.12 cm) (p = 0.007). The shorter semitendinosus graft length in the IB group may reflect natural anatomical variability rather than technique modification; the final prepared graft dimensions were similar. The mean gracilis graft length in Group A and Group B was 25.20 ± 1.15 cm and 25.48 ± 0.96 cm, respectively, with no statistically significant difference between the two groups (p = 0.356). The mean length of the final hexafold graft was 8.30 ± 0.40 cm in Group A and 8.18 ± 0.35 cm in Group B, while the mean graft diameter was 7.50 ± 0.55 mm and 7.63 ± 0.64 mm, respectively, with no significant differences between the groups (p = 0.270 and p = 0.430, respectively) (Table 5).

**Table 5:** Length of graft harvested and hexafold graft dimensions *indicates p < 0.05 as statistically significant

Variable	Domain	Group A		Group B		p-value
		Mean	SD	Mean	SD	
Length of graft harvested	Semitendinosus	26.44	1.12	27.28	0.98	0.007*
	Gracilis	25.20	1.15	25.48	0.96	0.356
Hexafold graft	Length	8.30	0.40	8.18	0.35	0.270
	Diameter	7.50	0.55	7.63	0.64	0.430

The Lysholm score was higher in Group A compared to Group B at all time points assessed, including presurgery (29.76 ± 7.45 vs. 29.12 ± 8.89), at 6 weeks (56.08 ± 6.25 vs. 55.88 ± 7.07), and at 12 weeks (88.24 ± 4.80 vs. 86.12 ± 4.93). However, these differences were not statistically significant (p = 0.784, 0.916, and 0.130, respectively) (Table 6).

**Table 6:** Lysholm score at presurgery and at 6- and 12-week follow-ups

Lysholm score	Group A		Group B		p-value
	Mean	SD	Mean	SD	
Presurgery	29.76	7.45	29.12	8.89	0.784
6 weeks	56.08	6.25	55.88	7.07	0.916
12 weeks	88.24	4.80	86.12	4.93	0.130

In Groups A and B, knee stiffness was observed in 8% and 12% of patients, pain in 4% and 0%, and swelling in 12% and 12%, respectively, with no significant difference (Table 7).

**Table 7:** Complications observed in the Group A and Group B

Complications	Group A		Group B		p-value
	N	%	N	%	
Knee stiffness	2	8	3	12	0.548
Pain	1	4	0	0	
Swelling	3	12	3	12	

## Discussion

Graft healing occurs in three phases: an initial stage with central necrosis and hypocellularity; a proliferative stage marked by peak cellular activity, remodeling, and revascularization; and a final ligamentization phase, where the graft transforms into a structure resembling the native ACL. During the proliferation stage, the graft is biomechanically weakest and most prone to failure. IB may help stabilize the graft during this critical window, supporting recovery [14]. The IB, or suture tape, acts as a scaffold, reinforcing the ACL graft and facilitating soft tissue healing. By offering additional mechanical support, it enables earlier and more confident mobilization, potentially accelerating rehabilitation and improving outcomes [15]. Postoperative rehabilitation is vital for restoring knee biomechanics. Typically initiated two weeks post-ACLR, early mobilization enhances tissue healing, collagen remodeling, and functional recovery. Structured rehab helps patients regain strength, promotes long-term joint health, and supports a safe return to activity [16].

The mean age in our study was significantly higher in Group A (32.92 ± 10.91 years) compared to Group B (26.56 ± 8.66 years). This aligns with existing literature showing that ACLR is common in the third decade of life. For instance, Vermeijden et al. reported a mean age of 29.4 ± 11.1 years [17], Szakiel et al. reported 31.53 ± 8.37 years [18], and Novaretti et al. noted a mean of 32.7 ± 11.4 years [19]. In contrast, Murray et al. observed a younger cohort, with a mean age of 17 years [20], while Daniel et al. found a median age of 19 in both IB and non-IB groups [21]. Toker et al. reported similar ages in isolated ACL (30.1 ± 4.1 years) and ACL with IB (28.1 ± 2.9 years) groups [22].

Our study showed a male predominance in both groups, with males comprising 60% in Group A and 84% in Group B. This trend aligns with Vermeijden et al., in which 59% of ACLR patients were male [17]. Other studies, including Murray et al. [20], Szakiel et al. [18], Toker et al. [22], Daniel et al. [21], and Novaretti et al. [19], reported either balanced gender distribution or no significant difference in sex ratios between groups, supporting the current findings. Overall, the gender distribution in our study reflects previously observed patterns, with no statistically significant variation across groups.

In our study, the most common mode of injury in both Group A and Group B was slip and fall (60% and 52%, respectively). Other modes of injury included fall from a vehicle (32% and 16%), vehicle collision (8% and 16%), trauma (0% and 8%), and twisting of the knee (0% and 8%). In comparison, Murray et al. reported that the majority of injuries in their study were non-contact, with all but one injury occurring during sports activities [20].

In this study, ACL injuries were similar across groups: complete (56% vs. 52%), full-thickness (24% each), and partial tears (4% vs. 16%). Avulsion (4%) and grade 2 tears (8%) occurred only in Group A. All PCLs were intact. The medial meniscus was intact in most cases (72% in Group A and 68% in Group B), with comparable rates of posterior horn and other tear types. Lateral meniscus integrity was also similar (76% vs. 72%), with minor differences in tear patterns. Arthritic changes were more frequent in Group B (56% vs. 36%) but were not statistically significant. Loose bodies (4%) and effusion (4%) were rare. These findings align with Murray et al., who reported similar rates of effusion and meniscal tears and noted preserved ACL remnants and a non-surgically managed MCL tear [20].

In our study, the mean semitendinosus graft length was significantly longer in Group B (27.28 ± 0.98 cm) than in Group A (26.44 ± 1.12 cm). The prepared graft length was similar between groups (25.20 ± 1.15 cm in Group A vs. 25.48 ± 0.96 cm in Group B). Hexafold graft length and diameter were comparable (length: 8.30 ± 0.40 cm vs. 8.18 ± 0.35 cm; diameter: 7.50 ± 0.55 mm vs. 7.63 ± 0.64 mm). Comparable findings were reported by Toker et al. (8.1 ± 0.6 mm vs. 8.4 ± 0.4 mm), Daniel et al., and Novaretti et al. (9.10 ± 0.87 mm vs. 8.82 ± 0.80 mm), with no significant differences in graft size between IB and non-IB groups [19,21,22].

In our study, Lysholm scores were slightly higher in Group A than in Group B at all time points - pre-surgery (29.76 ± 7.45 vs. 29.12 ± 8.89), 6 weeks (56.08 ± 6.25 vs. 55.88 ± 7.07), and 12 weeks (88.24 ± 4.80 vs. 86.12 ± 4.93) - though the differences were not statistically significant. This suggests that ACLR with IB yields comparable functional outcomes. The minimal clinically important difference (MCID) for Lysholm after ACLR has been reported to range from approximately 10-15 points; therefore, the mean between-group differences (~2 points) were below clinical relevance. Toker et al. similarly reported no significant difference in Lysholm scores between isolated ACLR (73.5 ± 5.8) and ACLR with IB (72.3 ± 6.1) [22]. Novaretti et al. observed marked postoperative improvement in both groups, without intergroup significance [19]. Bodendorfer et al., however, found that IB augmentation was linked to improved patient-reported outcomes, faster return to sport, and greater recovery to preinjury levels [23].

Postoperative complications were comparable: stiffness occurred in 8% (Group A) and 12% (Group B), pain in 4% and 0%, and swelling in 12% of both groups. Robertson et al. reported a 12% stiffness rate at six months, consistent with our findings [24].

This study has several limitations. First, the sample size was relatively small, and all participants were recruited from a single center, which may limit generalizability. Group allocation was based on patient preference rather than randomization, introducing potential selection bias. In addition, there was a significant baseline age difference between the two groups, which may act as a confounder in interpreting functional outcomes. Follow-up was limited to 12 weeks; therefore, the findings reflect only short-term patient-reported functional recovery and cannot assess graft healing, reinjury risk, or long-term stability. The study relied exclusively on a single patient-reported outcome measure (Lysholm), without objective measures such as instrumented laxity testing, return-to-sport rates, or radiologic or clinical assessment of graft integrity. Future research with larger, multicenter cohorts and longer follow-up, ideally using randomized allocation and comprehensive objective outcome measures, is warranted to validate and expand upon these preliminary findings.

## Conclusions

This study compared ACLR with and without IB. Group A (with bracing) had an older average age, and ACL tears were seen in all Group B patients and 84% of Group A patients; some Group A cases had associated avulsion or meniscal injury. Slip and fall was the most common mode of injury. Anterior drawer tests were positive in all cases, while Lachman and McMurray tests varied between groups. Most ACL injuries were complete or full-thickness, and all PCLs were intact. Group B had slightly longer harvested grafts, although final graft dimensions were similar. Lysholm scores were numerically higher in Group A at all follow-up points, but these differences were not statistically significant. Minor complications, such as pain, swelling, and stiffness, were comparable between groups. Overall, IB produced similar short-term functional outcomes without an apparent increase in early complications. Larger, multicenter studies with longer follow-up, randomized allocation, and objective stability measures are required to determine whether IB offers any meaningful clinical advantage over standard ACLR.
